# Supplementary material for: Prevalence and Genetic Characteristics of Avian Chlamydia in Birds in Guangxi, Southwestern China
Source: Microorganisms. 2025 Sep 22;13(9):2220. doi: 10.3390/microorganisms13092220 (PMC12472478; doi:10.3390/microorganisms13092220)
Supplement: Supplementary file 1 [file microorganisms-13-02220-s001.zip › Supplementary Materials Table S3.pdf]

Table S3. The similarities of nucleotide sequence of *ompA* gene in samples from waterfowl and human.

Strain	①The *ompA* gene sequence similarities with that of P5 strain %	②The *ompA* gene sequence similarities with that of D121 strain %
BSTLY12	68.9	66.3
BSTLY9	69.2	65.5
GLCY41	69.4	67.4
GLLYY1	98.5	98.3
GLLYY2	98.3	97.8
GLLYY3	98.5	98.3
HPDY1	98.8	98.3
HPDY2	98.4	98.1
HPDY3	98.8	98.3
LZY3	99.9	100
LZY37	99.1	99.0
LZY38	99.9	100
LDE5	99.3	99.0
LDE7	99.3	99.0
LDE11	99.4	99.3
NNE4	99.3	99.0
NNE12	99.8	99.8

① P5 strain: a *Chlamydia psittaci* isolate obtained from human in Shandong province, China; ② D121 strain: a *Chlamydia psittaci* isolate obtained from duck in Shandong province, China
